# Supplementary material for: Diffuse Optical Spectroscopy Assessment of Resting Oxygen Metabolism in the Leg Musculature
Source: Metabolites. 2021 Jul 29;11(8):496. doi: 10.3390/metabo11080496 (PMC8400025; doi:10.3390/metabo11080496)
Supplement: Supplementary file 1 [file metabolites-11-00496-s001.zip › metabolites-1324266-supplementary.pdf]

Supplementary Materials:

Table S1. Correlation statistics for all source-detector separations, including Pearson's R, concordance correlation coefficient, and associated p-value. The linear model used to describe the relationship between the NIRS-only arterial occlusion measure (VO_{2AO}) and the NIRS-only venous occlusion measure (VO_{2VO}) is described by the slope (standard error) and intercept (standard error).

	**Source-detector separation**			
	3.5 cm	3 cm	2.5 cm	2 cm
VO_{2VO} vs. VO_{2i}	$R^2 = 0.16$<br>p = 0.021	$R^2 = 0.052$<br>p = 0.202	$R^2 = 0.056$<br>p = 0.184	$R^2 = 0.024$<br>p = 0.390
VO_{2AO} vs. VO_{2VO}	$R^2 = 0.31$<br>p < 0.001<br>CCC = 0.40<br>Slope = 1.01(0.26)<br>Intercept = 0.01(0.01)	$R^2 = 0.19$<br>p = 0.008<br>CCC = 0.32<br>Slope = 0.79(0.27)<br>Intercept = 0.02(0.01)	$R^2 = 0.15$<br>p = 0.021<br>CCC = 0.25<br>Slope = 0.75(0.31)<br>Intercept = 0.02(0.01)	$R^2 = 0.09$<br>p = 0.075<br>CCC = 0.21<br>Slope = 0.51(0.28)<br>Intercept = 0.02(0.01)
VO_{2AO} vs. VO_{2i}	$R^2 = 0.33$<br>p < 0.001	$R^2 = 0.33$<br>p < 0.001	$R^2 = 0.28$<br>p = 0.002	$R^2 = 0.27$<br>p = 0.002
BF vs. BFI	$R^2 = 0.61$<br>p < 0.001	$R^2 = 0.58$<br>p < 0.001	$R^2 = 0.61$<br>p < 0.001	$R^2 = 0.62$<br>p < 0.001

Table S2. Repeatability of oxygen metabolism and blood flow measurements for all source-detector separations. Intraclass correlation coefficients (ICC) and coefficient of variation (CV) for oxygen metabolism measured during arterial occlusion and venous occlusion (VO_{2AO} and VO_{2VO}, respectively), as well as oxygen metabolism index (VO_{2i}), blood flow measured during venous occlusion (BF), and blood flow index (BFI). Note that only one source-detector separation was used for BFI (2.5 cm).

**Source-detector Separation**		**VO_{2AO}**	**VO_{2VO}**	**VO_{2i}**	**BF**	**BFI**
3.5 cm	ICC	0.88	0.85	0.77	0.83	NA
	CV	0.19 ± 0.13	0.24 ± 0.13	0.24 ±0.09	0.27 ± 0.12	NA
3 cm	ICC	0.81	0.84	0.76	0.85	NA
	CV	0.24 ± 0.18	0.27 ± 0.18	0.24 ± 0.10	0.29 ± 0.13	NA
2.5 cm	ICC	0.69	0.77	0.77	0.85	0.62
	CV	0.31 ± 0.22	0.31 ± 0.22	0.24 ± 0.10	0.32 ± 0.16	0.25 ± 0.11
2 cm	ICC	0.68	0.78	0.78	0.85	NA
	CV	0.34 ± 0.20	0.31 ± 0.20	0.25 ± 0.10	0.32 ± 0.23	NA
